# HIPPIE: Integrating Protein Interaction Networks with Experiment Based Quality Scores

**DOI:** 10.1371/journal.pone.0031826

**Published:** 2012-02-14

**Authors:** Martin H. Schaefer, Jean-Fred Fontaine, Arunachalam Vinayagam, Pablo Porras, Erich E. Wanker, Miguel A. Andrade-Navarro

**Affiliations:** 1 Max Delbrück Center for Molecular Medicine, Berlin, Germany; 2 Department of Genetics, Harvard Medical School, Boston, Massachusetts, United States of America; 3 IntAct Scientific Curator, EMBL-EBI, Wellcome Trust Genome Campus, Hinxton, Cambridge, United Kingdom; University Of Oxford, United Kingdom

## Abstract

Protein function is often modulated by protein-protein interactions (PPIs) and therefore defining the partners of a protein helps to understand its activity. PPIs can be detected through different experimental approaches and are collected in several expert curated databases. These databases are used by researchers interested in examining detailed information on particular proteins. In many analyses the reliability of the characterization of the interactions becomes important and it might be necessary to select sets of PPIs of different confidence levels. To this goal, we generated HIPPIE (Human Integrated Protein-Protein Interaction rEference), a human PPI dataset with a normalized scoring scheme that integrates multiple experimental PPI datasets. HIPPIE's scoring scheme has been optimized by human experts and a computer algorithm to reflect the amount and quality of evidence for a given PPI and we show that these scores correlate to the quality of the experimental characterization. The HIPPIE web tool (available at http://cbdm.mdc-berlin.de/tools/hippie) allows researchers to do network analyses focused on likely true PPI sets by generating subnetworks around proteins of interest at a specified confidence level.

## Introduction

Protein function occurs or is regulated by protein interactions and therefore knowledge on the partners of a given protein can give us important information regarding its activity. For instance, specific protein-protein interactions (PPIs) can be involved in diseases (see e.g. [1]). PPIs can be evaluated by many experimental methodologies, which have hugely different degrees of confidence and different experimental set-ups. For instance, while yeast two hybrid (Y2H) identifies direct physical interactions between two proteins, mass spectrometry (MS) based datasets report components of protein complexes, which may or may not be in direct physical contact. In addition to experimental methods, computational methods propose protein interactions based, for example, on orthology, protein domains known to interact, co-expression and functional annotations [2], [3].

PPIs are collected in several databases that make the data and the evidence behind it easily accessible and allow different mechanisms to query and display the data [4], [5], [6], [7], [8], [9], [10]. These resources are very useful for researchers interested in checking a small number of particular proteins of interest. However, PPI data can also be used globally for systematic network analyses, prediction of protein properties, and evaluation of novel datasets of PPIs produced in a high-throughput fashion.

Computational use of PPI datasets often requires selecting PPIs at particular levels of confidence. For example, the quality of a novel PPI dataset may be evaluated by its overlap with known interactions defined with high reliability, whereas a statistical analysis might require a large number of interactions therefore benefiting from a less restricted set of PPIs. The flexible selection of PPI datasets at various confidence levels requires a continuous scoring scheme for PPIs reflecting the reliability of their experimental characterization.

With the objective of creating a resource allowing the selection of PPIs by experimental confidence cut-offs, we generated HIPPIE (Human Integrated Protein-Protein Interaction rEference), a scored human PPI collection integrated from multiple sources. Following [8], we developed an expertly curated scoring scheme that takes into account the reliability of different experimental evidence in the definition of a PPI combining three types of information: experimental techniques used, number of studies finding the PPI, and reproducibility in model organisms.

A web tool to browse the data as well as the scored PPI dataset are provided at http://cbdm.mdc-berlin.de/tools/hippie. The scored dataset includes information on the data we used to build it so that modifications of the scoring mechanism can be easily achieved. We illustrate the usefulness of HIPPIE in increasing the coverage of novel PPI datasets and demonstrate that its scoring scheme reflects the reliability of the reported interactions.

## Methods

### 2.1 Sources

Interactions were retrieved from the following public databases: BioGRID (version 2.0.62; release date: March 16, 2010) [4], DIP (release date: December 30, 2009) [5], HPRD (version 8; release date: July 6, 2009) [6], IntAct (release date: March 29, 2010) [7], MINT (release date: 9 November 2009) [8], BIND (2004 release [11]), and MIPS (published: November 5, 2004) [10]. Genetic interactions were removed from BioGRID. Additionally, we integrated interactions from manually selected studies [12], [13], [14], [15], [16], [17], [18], [19], [20], [21], [22]; interactions from these studies were integrated that were not contained in the public databases at the time of integrating the sources. All resources integrated in HIPPIE are summarized in Table 1.

**Table 1:** PPI data sources integrated in HIPPIE.

PPI dataset	Reference	Size
HPRD	[6]	40110
BioGRID	[4]	30027
IntAct	[7]	28073
MINT	[8]	14094
Rual05	[19]	6946
Lim06	[17]	5579
Bell09	[13]	3300
Stelzl05	[20]	3232
DIP	[5]	1618
BIND	[11]	1415
Colland04	[21]	882
Lehner04	[16]	385
Albers05	[12]	290
MIPS	[10]	252
Venkatesan09	[22]	239
Kaltenbach07	[15]	227
Nakayama02	[18]	84
HIPPIE		72916

Where available, we retrieved the information on the originating study and the experimental methodology used to measure each interaction from the source databases and also assigned an experimental category to interactions from the additionally included studies. As a result, more than 99% of all interactions in HIPPIE are associated to at least one of the methods listed in Table 2 and are annotated with the studies in which they were detected.

**Table 2:** Scores for experiment types.

Technique	PSI	score	Technique	PSI	Score
3 hybrid method	MI:0588	5	footprinting	MI:0417	3
acetylation assay		7.5	FRET		6
Affinity Capture-Luminescence		5	gal4 vp16 complementation	MI:0728	5
Affinity Capture-MS		5	genetic interference	MI:0254	0
Affinity Capture-RNA		2	gst pull down	MI:0059	5
Affinity Capture-Western		5	gtpase assay	MI:0419	7.5
affinity chromatography technology	MI:0004	5	his pull down	MI:0061	5
affinity technology	MI:0400	5	homogeneous time resolved fluorescence	MI:0510	7
anti bait coimmunoprecipitation	MI:0006	5	imaging technique	MI:0428	1
anti tag coimmunoprecipitation	MI:0007	5	in vitro	MI:0492	1
antibody array	MI:0678	5	in vivo	MI:0493	1
array technology	MI:0008	3	in-gel kinase assay	MI:0423	7.5
atomic force microscopy	MI:0872	9	inferred by curator	MI:0364	1
beta galactosidase complementation	MI:0010	5	ion exchange chromatography	MI:0226	3
beta lactamase complementation	MI:0011	5	isothermal titration calorimetry	MI:0065	10
bimolecular fluorescence complementation	MI:0809	6	kinase homogeneous time resolved fluorescence	MI:0420	7.5
Biochemical	MI:0401	1	lambda phage display	MI:0066	6
Biochemical Activity		5	lex-a dimerization assay	MI:0369	5
bioluminescence resonance energy transfer	MI:0012	6	light microscopy	MI:0426	1
Biophysical	MI:0013	1	light scattering	MI:0067	10
blue native page	MI:0276	3	mammalian protein protein interaction trap	MI:0231	6
chromatin immunoprecipitation assay	MI:0402	2	mass spectrometry studies of complexes	MI:0069	5
chromatography technology	MI:0091	1	methyltransferase assay	MI:0515	7.5
circular dichroism	MI:0016	9	methyltransferase radiometric assay	MI:0516	7.5
classical fluorescence spectroscopy	MI:0017	7.5	molecular sieving	MI:0071	2
Co-crystal Structure		10	no experiment assigned		0
Co-fractionation		1	nuclear magnetic resonance	MI:0077	10
Co-localization		1	peptide array	MI:0081	5
Coimmunoprecipitation	MI:0019	5	phage display	MI:0084	6
colocalization by fluorescent probes cloning	MI:0021	1	phosphatase assay	MI:0434	7.5
colocalization by immunostaining	MI:0022	1	phosphotransfer assay		7.5
colocalization/visualisation technologies	MI:0023	1	polymerization	MI:0953	5
comigration in gel electrophoresis	MI:0807	3	protease assay	MI:0435	7.5
comigration in non denaturing gel electrophoresis	MI:0404	3	protein array	MI:0089	5
comigration in sds page	MI:0808	3	protein complementation assay	MI:0090	5
competition binding	MI:0405	5	protein cross-linking with a bifunctional reagent	MI:0031	5
confocal microscopy	MI:0663	1	protein kinase assay	MI:0424	7.5
Copurification	MI:0025	2	protein tri hybrid	MI:0437	5
Cosedimentation	MI:0027	2	Protein-peptide		5
cosedimentation in solution	MI:0028	2	Protein-RNA		0
cosedimentation through density gradient	MI:0029	2	pull down	MI:0096	2.5
cross-linking study	MI:0030	5	pull-down/mass spectrometry		5
deacetylase assay	MI:0406	7.5	Reconstituted Complex		10
demethylase assay	MI:0870	7.5	reverse phase chromatography	MI:0227	1
dihydrofolate reductase reconstruction	MI:0111	6	reverse two hybrid	MI:0726	5
dynamic light scattering	MI:0038	9	ribonuclease assay	MI:0920	7.5
electron microscopy	MI:0040	5	saturation binding	MI:0440	7.5
electron paramagnetic resonance	MI:0042	9	scintillation proximity assay	MI:0099	7.5
electron tomography	MI:0410	9	solid phase assay	MI:0892	1
electrophoretic mobility shift assay	MI:0413	2	surface plasmon resonance	MI:0107	10
electrophoretic mobility supershift assay	MI:0412	2	t7 phage display	MI:0108	6
enzymatic study	MI:0415	1	tandem affinity purification	MI:0676	5
enzyme linked immunosorbent assay	MI:0411	5	tox-r dimerization assay	MI:0370	5
experimental interaction detection	MI:0045	1	transcriptional complementation assay	MI:0232	5
far western blotting	MI:0047	5	transmission electron microscopy	MI:0020	5
filamentous phage display	MI:0048	6	two hybrid fragment pooling approach	MI:0399	5
filter binding	MI:0049	5	Two-hybrid	MI:0018	5
fluorescence correlation spectroscopy	MI:0052	10	ubiquitin reconstruction	MI:0112	5
fluorescence microscopy	MI:0416	1	x ray scattering	MI:0826	9
fluorescence polarization spectroscopy	MI:0053	10	x-ray crystallography	MI:0114	10
fluorescence technology	MI:0051	1	x-ray fiber diffraction	MI:0825	9
fluorescence-activated cell sorting	MI:0054	1	yeast display	MI:0115	5
fluorescent resonance energy transfer	MI:0055	6			

To add to the confidence scoring of experimentally verified human PPIs a component based on experimental evidence in non-human organisms we included data from three databases that map interactions between non-human protein pairs to their human orthologs: HomoMINT (release date: March 5, 2009) [23], I2D (release date: January 7, 2010) [2] and the PPI dataset from [24].

### 2.2 Identifier mapping

Different public PPI databases and datasets use different types of gene or protein identifiers. We aimed at mapping all protein pairs listed in HIPPIE to Entrez Gene and UniProt identifiers. For this purpose we applied the database identifier mapping tables curated by UniProt [25] and the HUGO Gene Nomenclature Committee (HGNC) [26]. We mapped all database entries to their canonical representatives and did not consider splicing forms. In the web interface the data can be queried either by protein (UniProt id or accession) or by gene identifier (Entrez Gene id or gene symbol). Interactions containing identifiers that could not be mapped to human Entrez Gene ids or UniProt ids were not included in HIPPIE.

Mapping PPIs to the genes encoding the interacting proteins is affected by certain ambiguity since the same protein sequence may be encoded by duplicated genomic loci. In the flat file version of HIPPIE these ambiguous PPIs are expanded such that a given PPI is represented by all possible combinations of gene identifiers.

### 2.3 Score calculation

For each interaction a score *S* between 0 and 1 was calculated reflecting the reliability of its combined experimental evidence. This score was calculated as a weighted sum of three different subscores which are *s_s_* (a function of the number of studies in which an interaction was detected), *s_t_* (a function of the number and quality of experimental techniques used to measure an interaction; see below for details) and *s_o_* (a function of the number of non-human organisms in which an interaction was reproduced). Each of these three subscores *s_i_* was calculated with a non-linear saturating function of the form:
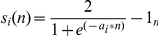
such that *s_i_(0)* = 0 and *s_i_(∞)* = 1, where the *a*_i_ are constants that control the steepness of the function.

For subscore *s_s_*, *n* is the number of different studies where the interaction was reported (number of PubMed identifiers associated), regardless of whether multiple experimental evidence was provided in each study.

For subscore *s_o_*, *n* is the number of species where orthologs of the interacting proteins could be defined and were found experimentally to interact (currently *Bos taurus*, *Caenorhabditis elegans*, *Canis familiaris*, *Drosophila melanogaster*, *Gallus gallus*, *Mus musculus*, *Rattus norvegicus*, *Saccharomyces cerevisiae*, and *Sus scrofa*).

For subscore *s_t_*, *n* is a sum of scores from different experimental techniques by which an interaction was verified (even if used in the same study). Most PPI databases use controlled vocabulary descriptors for these experimental techniques as defined by the PSI-MI ontology [27], however for some terms we could not find an equivalent ontology term. Manual curation was used to assign a score to each PPI detection method ranging from 0 (no experiment assigned, less than 1% of PPIs) to 10. Scores and corresponding PSI-MI codes are displayed in Table 2. Methods that can ascertain interactions with the highest reliability, such as in vitro techniques like X-ray crystallography, were assigned the highest scores. Complementation-based assays and affinity based technologies were roughly equally scored with an average value of 5, slightly increased for those methods that are used generally in homologous, more physiological setups, such as FRET. Methodologies that do not directly provide evidence for interaction, such as colocalization or cosedimentation, are scored with the lowest values. The total score *S* was calculated as a weighted sum of the three subscores:
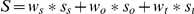
with 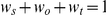.

It is important to note that our dataset does not include interactions not experimentally verified with human proteins: no interaction received a score alone from its verification in non-human organisms. We also remark that this scoring scheme does not consider computational evidence other than the definition of orthology relations from human proteins to proteins in other organisms.

### 2.4 Parameter selection

The six free parameters of the scoring formula (*a_s_*, *a_o_*, *a_t_*, *w_s_*, *w_o_* and *w_t_*) were optimized by performing a grid search in the parameter space. We performed the search in the range [0, 3] for the *a_i_* and in the range [0, 1] for the *w_i_*. We chose a step width of 0.1 for both *a_i_* and the *w_i_*. The step width was chosen sufficiently small such that selecting neighboring parameter combinations resulted only in small changes in the interaction scores which decreased the probability of missing an optimal solution. Constraints were set on the weights *w_i_* by requiring that they sum up to 1. Parameter combinations leading to only few discrete scores were excluded (this happened, for example, when *w_t_* was set to 0, since the different experimental weights account for a large fraction of the score's granularity).

PPIs are sometimes reported in multiple studies. We reasoned that we could use this property to assess the performance of a parameter combination. To do this evaluation we used the IntAct dataset, which currently consists of 28 073 interactions (38.5% of HIPPIE). This dataset has explicit associations between studies and experiments, and the experimental information is annotated following the PSI-MI format.

The assessment of performance of a parameter set was done by successively removing each one of the 109 studies in IntAct that contain at least 10 interactions and more than two PPIs found in multiple studies. For each study j, we recalculated the scores of the remaining dataset, IntAct_red_, found the set of PPIs described both in the study *j* and in IntAct_red_, 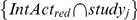, and computed the deviation from random expectation of the number of highly scored interactions among the overlap:
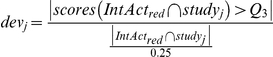
where *Q_3_* is the upper quartile of the score distribution of IntAct_red_.

To measure the overall performance of a parameter combination we chose a function *f* of the weighted mean of the logarithm of dev_i_ over all studies:
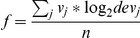
where the weights *v_i_* were chosen proportional to the overlap size between IntAct_red_ and study_j_ and n is the number of studies. The best parameter combination maximizes *f*.

We found several parameter combinations (several thousand optimal combinations out of more than 700 000 different parameter combinations tested) maximizing the function *f* (max(*f*) = 1.023). From the equally well performing parameter combinations we chose the set of parameters that resulted in the largest spread of the distribution of scored interactions. For that purpose the scores of the entire HIPPIE were repeatedly calculated for each of the optimal parameter combination and for each score distribution the interquartile range (*iqr*) was determined. We found that the parameter set [*a_s_* = 2.3, *a_o_* = 1.6, *a_t_* = 0.2, *w_s_* = 0.6, *w_o_* = 0.1, *w_t_* = 0.3] maximized both *f* and *iqr*.

## Results

HIPPIE is a dataset of experimentally measured human PPI derived from several publicly available PPI datasets (Table 1). For reference, we distribute a stable release of HIPPIE consisting of 72 916 interactions, which was used in this manuscript for several descriptive analyses (Table S1; HIPPIE version 1.2). The live version of HIPPIE is monthly updated making use of the web query interface PSICQUIC [28], which allows us to automatically retrieve the newest interaction data from most of the manually curated source databases (BioGrid, IntAct, MINT, DIP and BIND) and integrate the new interactions and updated evidence records into HIPPIE.

The network is accessible via a web tool (http://cbdm.mdc-berlin.de/tools/hippie) that allows for querying the interactions by a gene symbol, Entrez gene id or UniProt identifier (id and accession). On the result page a confidence score is listed with each interaction partner of the query protein and detailed information on the evidence contributing to the confidence score can be accessed. Links to the original studies are provided.

A typical problem after generation of experimental results producing a list of genes, proteins and/or interactions between them, is the evaluation of the results in relation to the known PPI data. For example, a researcher may have obtained proteomics data for a few proteins of interest and wants to evaluate the novelty of the interactions, or the possible relation of the interactors with a disease protein of interest.

To facilitate this analysis, HIPPIE can be queried with a set of proteins and/or interactions between them from which a network of known data around the proteins of interest is constructed. The online tool will identify interactions between the proteins submitted (layer 0 network), or their interactors not contained in the query set (layer 1 network). The computation of networks with more layers might be lengthy if hundreds of protein partners have to be analysed. For this we provide a Java command line tool (available from http://cbdm.mdc-berlin.de/tools/hippie and also deposited at the SourceForge open software archive: https://sourceforge.net/projects/hippiecbdm) that will do the computation on the local machine of the user for large input sets or neighbours of neighbours. A confidence threshold to control the reliability and size of the constructed network can be also applied. Additionally, we provide a filter option for the PSI-MI interaction type annotation provided by most of HIPPIE's source databases. This feature allows for selecting direct physical interactions from HIPPIE. The thereby generated HIPPIE subnetworks can be exported from HIPPIE for further analyses or can be visualized using the tool Cytoscape Web [29], which has been integrated into HIPPIE.

The web site also offers the entire HIPPIE dataset for download in two different formats: in PSI-MI TAB 2.5 format as defined by the Protein Standard Initiative [27] and in our own tab delimited flat file format. Currently we distribute a freeze version (version 1.2) used in this manuscript for analyses, and the monthly updated version.

While merging the different data sources we kept track of the information about which experimental system type was used to detect each single interaction and whether there were several studies where the interaction was found. Additionally we retrieved the interaction data from PPI databases that link interactions in non-human model organisms to their human orthologs. From these different types of information (experimental systems, number of studies and reproducibility in other organisms) we calculated an overall score reflecting the reliability of each interaction (See Methods for details and Table 2).

We note that the different experimental methodologies behind the PPIs in HIPPIE are able to detect direct physical interactions between proteins to a varying degree. Even though some of them are in fact measuring co-membership in larger protein-complexes we will refer to all types of associations detected by these methods as interactions or PPIs. The HIPPIE score tries to reflect both the reliability of the various methods as well as the ability to detect direct rather than indirect interactions.

**Figure 1:**
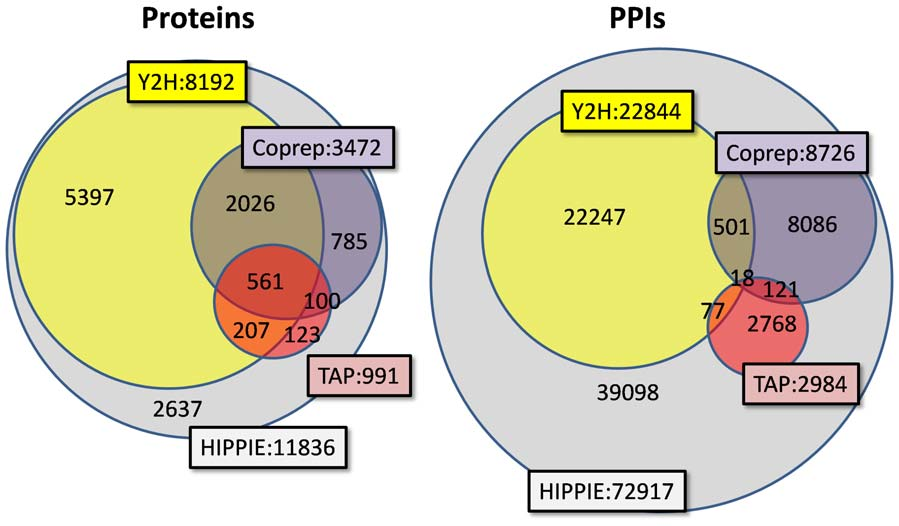
Coverage of HIPPIE and overlap by three technique specific datasets. Left: proteins. Right: PPIs. Y2H is yeast-two-hybrid, Coprep is anti-bait coimmunoprecipitation and MS is affinity capture mass spectrometry. The protein numbers show that Y2H can focus on many proteins that have not been targeted by the other two techniques. Together the three techniques already cover 80% of all proteins currently considered in HIPPIE (i.e. 80% of all proteins in HIPPIE participate in at least one Y2H, Coprep or MS experiment). However, the overlap in PPIs between these datasets and to the remainder of HIPPIE is much smaller indicating that PPI detection is technique specific. Nevertheless, one can appreciate that similar techniques have a bias towards detecting similar PPIs, here illustrated by the significant overlap between Coprep and MS and by the little overlap of Y2H to the other two techniques.

The number of PPIs derived from different experimental system types was very variable. HIPPIE integrates various datasets dealing with different experimental systems and thus contains a larger amount of interactions than each of those sets separately (Table 1). Values for three well populated and meaningful sources of PPIs, Y2H, anti-bait coimmunoprecipitation (Coprep), and tandem affinity purification (TAP) are shown in Figure 1 that cover 78% of the total amount of proteins in the current version of HIPPIE, but only around 50% of its interactions. Coprep and TAP share relatively many PPIs between each other (139 PPIs) compared to the other pairwise overlaps between methods. For example, TAP shares 95 interactions with Y2H despite the much higher amount of Y2H interactions as compared to Coprep. This higher overlap between Coprep and TAP in comparison with the Y2H data might reflect the similarity between the first two approaches in comparison with the latter, as Coprep and TAP are both based on antibody capture of a protein complex while Y2H is based on the reconstitution of a binary interaction inside of a heterologous system (yeast).

To illustrate the benefit of using a large dataset such as HIPPIE, we compared it with novel high-throughput PPI datasets not used for its production. We chose two high-throughput PPI datasets from the recent literature: a Y2H dataset, Y2He [30], containing 551 PPIs between 434 proteins and a MS dataset, MSe [31], containing 711 PPIs between 424 proteins. The coverage of the Y2He and MSe datasets by HIPPIE was of 120 (21.8%) and 73 (10.3%) PPIs, respectively.

**Figure 2:**
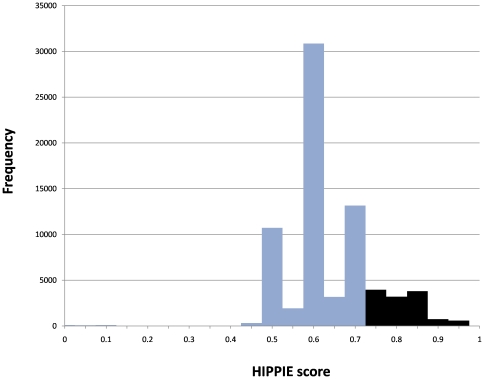
Distribution of HIPPIE confidence scores. Interactions with scores above 0.73 (black bars) constitute only 25% of all and could be considered high-confidence interactions. According to the design of the scoring function, such score implies that the interaction is supported by multiple evidence.

We evaluated the usefulness of the HIPPIE score using the two novel datasets. The HIPPIE database was divided in a high quality subset containing the top 25% highest scoring interactions (score > = 0.73) and a lower quality subset (score <0.73; see Figure 2). Then, we compared the fraction of PPIs in each HIPPIE subset that was recalled by the novel dataset. If the scores are meaningful one would expect better recall of the set with high-confidence scores.

To measure the recall of HIPPIE by an external dataset of PPIs one has to consider that some HIPPIE PPIs may not be detectable by the experimental setup used to produce the external dataset. In the case of Y2H and MS datasets a number of proteins are used as baits. Therefore, we considered for each of these studies that the “detectable PPIs” from HIPPIE were those where at least one of the interacting proteins was a bait in the study considered (Table 3). The values of detectable PPIs and recall were used to calculate one-sided Fisher's exact tests to assess the significance of the differences in recall between high and low confidence HIPPIE subsets. The high quality subset had the largest overlaps in percentage with the PPIs of the novel datasets and these overlaps were significant (Table 3; p-values of 6.40e-15 and 1.75e-6 for Y2He and MSe, respectively) suggesting that the PPI score correlates with experimental reproducibility.

**Table 3:** Coverage of HIPPIE by novel datasets.

HIPPIE subset	HIPPIE subset size	Y2He		MSe	
		detectablePPIs	Overlap PPIs(recall)	detectablePPIs	Overlap PPIs (recall)
score > = 0.73	18592	2239	75 (3.3%)	322	41 (12.7%)
score <0.73	54324	5760	45 (0.8%)	806	32 (4.0%)

## Discussion

In this work we presented HIPPIE, an integrated dataset of human protein interaction data scored according to experimental evidence. This resource has been created for those researchers that need to use globally the complete knowledge on human protein interactions. This is required in systems biology studies and in the evaluation of high-throughput results (e.g. novel PPI datasets) that require contrasting results with interactions selected for a particular level of reliability.

HIPPIE currently integrates 72 916 interactions from several public PPI resources scored according to confidence. For comparison, the complete human interactome map has been estimated to contain between 200 000 and 400 000 interactions (according to [32] and [33], respectively) suggesting that our knowledge of the human interactome is still incomplete. Nevertheless, producing a large collection of integrated PPI data is critical for its usability because novel high-throughput PPI datasets often contain just hundreds of PPIs and might have little overlap with smaller existing PPI resources integrated in HIPPIE.

Several resources have been created that, like HIPPIE, integrate PPI data from multiple sources but do not have a focus on distributing a simple scored dataset, while offering excellent tools to examine evidence behind each PPI (e.g. iRefWeb [34]) or do not focus on experimentally verified interactions (e.g. STRING [35]). Some other databases offer a continuous confidence scoring scheme, e.g. MINT [8] and HAPPI [36], but they do not allow batch scoring of PPI sets or the exclusive retrieval of high confidence interactions and lack the integration of several important high-throughput experimental datasets. The scoring system of MINT is closer to the one we use as it considers levels of technical evidence, number of studies and orthology [8]; however, as the PPI data from MINT is manually curated, the amount of human PPIs in MINT is currently less than a third of those in HIPPIE, limiting its use in the evaluation of novel datasets. Finally, in contrast with MINT and HIPPIE, HAPPI contains only a small fraction of PPIs experimentally derived in human while the majority are either computationally predicted or inferred from other species.

We are aware that any assignment of reliability scores to experimental techniques necessarily reflects the individual belief of researchers. We tried however to base our selection of parameters and weights in the scoring formula on objective criteria by optimizing the performance of our scoring scheme in assigning high values to reproducible interactions. For researchers who nevertheless wish to modify either the selected parameters or the scores assigned to the different techniques we offer a tool at our homepage that allows the scoring of HIPPIE using an altered set of these values.

HIPPIE has been used for the evaluation of existing novel PPI datasets showing that it increases their coverage over individual resources and that its scoring scheme correlates with the ability to find a PPI in experimental data not included in the database (Table 3). A web tool to query the data, the scored PPI dataset as well as the raw data are available at http://cbdm.mdc-berlin.de/tools/hippie. The tool allows batch annotation of datasets of PPIs. Future work on HIPPIE will be directed towards the inclusion of novel datasets and versions for major model organisms.

## Supporting Information

Table S1 **Scored dataset of PPIs.** The columns indicate (1) UniProt identifier and (2) Entrez Gene identifier of the first protein partner, (3) UniProt identifier and (4) Entrez Gene identifier of the second protein partner, (5) score and (6) a comment field summarizing the origin of the evidence. Evidence is arranged in three types: experiments, pmids, and sources. Experiment types are indicated in Table 2. Pmids are the PMID of manuscripts reporting the interaction. Sources are the datasets where the interaction was found and are indicated in Table 1. Multiple evidences for each type are separated by semicolon and multiple evidence codes for each type are separated by comma. If one protein maps to several genes, each combination of genes is listed in a separate line. This table is available from: http://cbdm.mdc-berlin.de/tools/hippie/hippie_v1_2.txt.
(TXT)
